# Use of nonaversive handling and training procedures for laboratory mice and rats: Attitudes of American and Canadian laboratory animal professionals

**DOI:** 10.3389/fvets.2022.1040572

**Published:** 2022-12-09

**Authors:** Carly I. O'Malley, Raina Hubley, Carly Moody, Patricia V. Turner

**Affiliations:** ^1^Global Animal Welfare and Training, Charles River Laboratories, Wilmington, MA, United States; ^2^Department of Pathobiology, University of Guelph, Guelph, ON, Canada

**Keywords:** low stress handling, attitudes, rats, mice, human-animal interactions

## Abstract

Nonaversive or low stress handling techniques can reduce fear and stress in research rodents, ultimately improving study data quality. Uptake of low stress handling has been slow in the USA and Canada. In this study we explored the understanding, experience, and attitudes toward low stress handling of rats and mice in laboratory animal professionals from the USA (US) and Canada (CA). Participants (*n* = 40) were recruited for a standardized interview and job categories were divided into veterinary/PhD level roles (doctoral level; DL) and non-veterinary/non-PhD level roles (non-doctoral level, NDL) (US: 23, DL: 9, NDL: 14; CA: 17, DL: 8, and NDL: 9). Interviews were transcribed and analyzed using NVIVO. Two research assistants independently coded themes for each question and consolidated responses based on commonality. Laboratory animal professionals understood the benefits of low stress handling and training techniques with rats and mice, stating reduced stress, better data, and improved welfare, with CA participants more likely to mention animal welfare as a benefit, and DL more likely to mention improved research data and reduced stress. Participants across demographic groups indicated improved job satisfaction and decreased stress as the positive impacts low stress handling would have on their positions. The primary perceived barriers to low stress handling implementation were researcher attitudes, the time needed to implement and use these techniques, and training personnel to use the techniques properly and consistently. To promote refinement of handling of rats and mice, more educational opportunities on the benefits and implementation of low stress handling techniques need to be provided to laboratory animal professionals, as well as to researchers.

## Introduction

Human-animal interactions occur frequently in a research environment related to husbandry and experimental activities, and contribute greatly to overall animal welfare outcomes (1, 2). Frequent interactions that are perceived as negative by animals can lead to chronic stress that may disrupt physiologic and behavioral processes, resulting in fear toward people, and increasing injury risk for the animal and handler (3–5). Improving handling methods can help reduce negative responses during human-animal interactions, reduce external influences on study data, and optimize animal health and welfare (1, 3, 4). Positive human-animal interactions and animal cooperation can be promoted through use of nonaversive or low stress handling methods, habituation, and positive reinforcement training (3, 4). Low stress handling specifically refers to patient, behavior-centered handling techniques that emphasize minimal restraint when working with animals. Low stress handling programs can also include training techniques such as habituation, defined as reducing the fear response through repeated exposure to a stimulus (5, 6), and positive reinforcement training, which increases the likelihood of a desired behavior through provision of a reward (6).

Rats and mice are common species worked with in biomedical research (7), but despite this, low stress handling, habituation, and training techniques are not commonly practiced compared to other species such as dogs (8, 9) and primates (10). For mice and rats, low stress handling primarily refers to the way in which animals are removed from and placed into their holding enclosures, and includes tunnel or cup/body handling (11, 12). For mice, there is a large body of evidence demonstrating that tail handling is aversive and results in stress and anxiety, which is reflected in physiologic measures such as increased plasma corticosterone (12, 13) and blood glucose levels (12), and reduced breeding efficiency (14). There are also altered behavioral responses, including increased anxiety as evidenced in elevated plus maze (12, 15, 16) and open field tests (12, 16, 17), reduced voluntary human interaction test responses (3, 15–18), changes in habituation-dishabituation tasks (19), and altered response to rewards (16, 18). The evidence also highlights tunnels as the preferred method of handling for mice (15), and that as little as 2s is needed to habituate mice to tunnel handling (3), and the effects of low stress handling remain even when tail handling is subsequently used for restraint for procedures (3, 20). There is less research investigating preferred handling methods for rats, but there is evidence that rats also find tail handling aversive (21) and that habituating rats to handling and/or using gentle handling is beneficial in reducing anxiety-like behaviors (22, 23), depressive-like states (23), improving learning and memory (22), reducing variation in behavior tests (24), and improving human-animal interactions (25). There is also evidence that implementing nonaversive restraint techniques for rats reduces stress-related behaviors (i.e., struggling, vocalizations, and defecation) and plasma corticosterone levels compared with tail handling restraint techniques (26).

Refinement is one of the 3Rs, and is a key ethical principle driving improvements in research animal care and welfare, the aim of which is to minimize pain and distress in research animals (27). With ample evidence demonstrating that tail handling is aversive to mice and rats, adoption of low stress handling techniques should be a priority. Unfortunately, despite this body of evidence there is still slow uptake of these techniques with handlers using tail handling regularly (28). To better understand why this is, the goal of this study was to explore the understanding, experience, and attitudes toward low stress handling and training techniques (habituation and positive reinforcement training) with rats and mice with laboratory animal professionals within the USA and Canada. The aims of this work were to understand the motivations and impediments to refining handling techniques for rats and mice, and to compare attitudes between countries (USA vs. Canada) and job categories (veterinary and PhD-level professionals vs. those in more technical roles).

## Methods

All methods were approved by the University of Guelph Research Ethics Board (REB#: 20-06-022).

Participants were recruited between December 2020 to April 2021 using email advertisements sent to professional membership listserves including the American Association for Laboratory Animal Science; AALAS, Canadian Association for Laboratory Animal Science; CALAS, and Canadian Association for Laboratory Animal Medicine; CALAM. The advertisement asked those interested to contact the researchers. Inclusion criteria for participation were that participants had to be 18 years of age or older, and currently working in a laboratory animal facility with laboratory mice and rats in Canada or the USA.

Participants were contacted to set up an interview, and the consent transcript was emailed. Once the date and time were confirmed, the researchers sent a link to the virtual meeting held *via* Microsoft Teams (Microsoft Corporation, Redmond, WA, USA). During the interview, the interviewer read the consent transcript aloud and asked for verbal consent to participate. Interviews took approximately 30–45 min and were audio-recorded. Participants were asked 16 open-ended questions (see Supplementary Interview Questions) and shown two demonstration videos of nonaversive handling techniques (mice: https://www.youtube.com/watch?v=bdtVZtrr69c&feature=emb_logo; rats: https://www.youtube.com/watch?v=gbsz_LZwuCM&feature=emb_logo). After the interview, participants were sent a link to a demographic survey on SurveyMonkey (Momentive Inc, San Mateo, CA, USA) to collect data on country, region, job function, education level, age, gender, and type of employer. The demographic survey was optional, and participants could skip questions as they saw fit.

**Figure 1:**
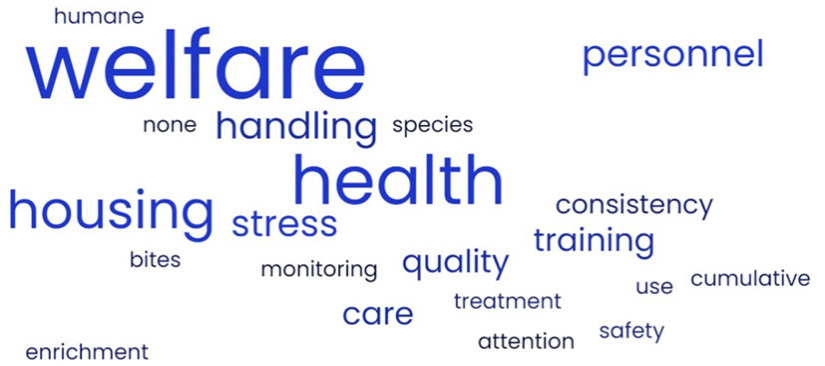
Word Cloud of the most significant day-to-day concerns of laboratory animal professionals working with research rats and/or mice.

**Figure 2:**
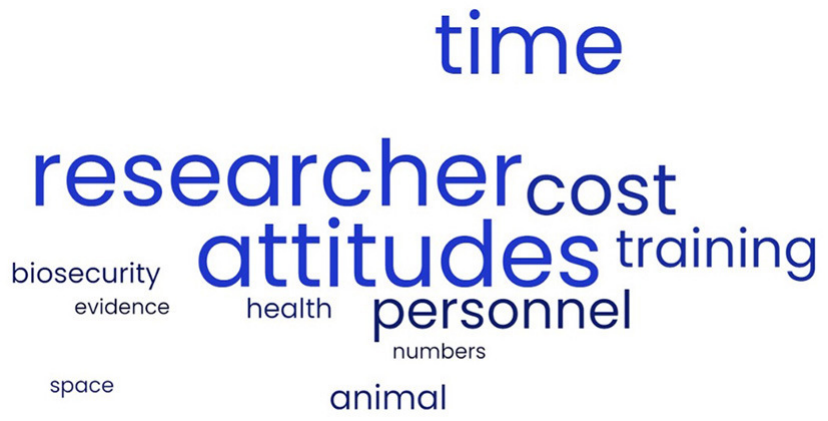
Word Cloud of perceived barriers of laboratory animal professionals to implementing low stress handling techniques with research rats and mice.

All interviews were transcribed by a single individual using validated methods (29). Transcripts were uploaded into NVIVO (QSR International Inc., Burlington, MA, USA) and independently coded for analysis by two of the researchers to identify themes and subthemes for each question. Each coder identified the top themes and subthemes based on how many participants mentioned it. Themes were compared between the coders. Initial consistency of major themes was high between the coders; therefore, discussions were based on confirming similar terminology and interpretation of responses, and consolidating results into one output with top themes and subthemes summarized by country [USA and Canada (CA)] and job category. Professions were grouped into veterinary and PhD-level roles (DL) and non-veterinary and non-PhD-level roles (e.g., in-life technicians; NDL). Both coders agreed on the final output. The word clouds in Figures 1 and 2 were generated by listing the total number of times a theme was coded per participant and inputting the list into Free Word Cloud Generator (https://www.freewordcloudgenerator.com/generatewordcloud; Salt Lake City, UT, USA).

## Results

### Demographic information

Forty participants completed interviews (US: 23, DL: 9, NDL: 14; CA: 17, DL: 8, and NDL: 9). Further demographic information for participants is presented in Table 1 (a total of 38 participants completed the demographic survey). The primary job categories included clinical or compliance veterinarians, veterinary technicians, animal care or welfare personnel, trainers or training coordinators, laboratory or facility managers, veterinary and animal care directors, technical services personnel, graduate students, and postdoctoral fellows working directly with mice or rats.

**Table 1:** Demographic information for interview participants (*n* = 40).

**Country**	**Position**	**Region**	**Highest level of education**	**Age**	**Gender**	**Institution type**
US: 23	DL: 9 NDL: 14	Northeast: 3 Midwest: 7 South: 7 West: 4	Secondary School: 0 Associate's certificate: 0 BA/BSc: 8 DVM/VMD: 8 MS, PhD: 5	18-30: 4 31-40: 10 41-50: 3 51-60: 3 > 60: 1	Male: 1 Female: 20	University/College: 10 Hospital: 3 Government: 2 Industry/CRO: 4 Other: 2
Canada: 17	DL: 8 NDL: 9	West: 5 Central: 11 Eastern: 0	Secondary School: 0 Associate's certificate: 4 BA/BSc: 1 DVM: 4 MS, PhD: 7	18-30: 2 31-40: 1 41-50: 6 51-60: 7 >60: 0	Male: 1 Female: 15	University/College: 12 Hospital: 3 Government: 0 Industry/CRO: 1 Other: 0

Demographic information was collected via survey which was optional and allowed participants to skip questions at will. A total of 38 participants completed the demographic survey.

DL, doctoral level; NDL, non-doctoral level, CRO, contract research organization.

### Concerns

Participants were asked what their biggest day-to-day concern was when working with research rats and mice. The concerns are summarized in Figure 1. The top concerns noted were animal welfare, animal health, training personnel, and stress. Housing and environment were additional significant concerns for US NDL and handling was a top concern for CA DL.

### Understanding of and attitudes toward techniques

The first set of questions asked participants about their understanding, experience with, and attitudes toward habituation and positive reinforcement training of mice and rats.

Understanding of the term habituation was low, with only 17.5% of participants providing the correct definition of repeated exposure to a stimulus. Most participants described “getting the animal used to” a situation or environment. Some participants described gradual exposure to a stimulus starting at low thresholds or providing a treat after a procedure. CA DL had a better understanding of habituation compared US DL and NDL from both countries, with 50% of this population providing the correct definition. Participants had a better understanding of the term positive reinforcement training than for habituation, with 60% of participants providing the correct definition, mentioning provision of a reward for a desired behavior. When describing positive reinforcement training, some participants also mentioned that it is training without punishment. US NDL had a better understanding of positive reinforcement training, with 71.4% of participants in this group providing the correct definition.

Half of the participants (50%) had no or minimal experience using these techniques with research rats and mice. Experience with habitation was primarily related to handling, but habituation to restraint, oral dosing, and use of respiratory/anesthetic cones was also mentioned. One key response from this question was:

“Particularly with rats… I've trained them to do basic skills, mostly target training. However, none of these have been implemented on a large scale.”

Therefore, even if participants were using habituation techniques with rats and mice, habituation wasn't widespread at their institution. When asked about their experience with these techniques, it was common for participants to mention the species in their response, to specify that they have used these techniques with other research species, such as large animals, but not with rodents, such as:

“I work with rodents and large animals, so sometimes you think of it more with large animals than with rodents. We don't give them a treat like we would with a large animal.”

Overall, US participants had less experience with these training techniques than CA participants.

The potential benefits to using habituation and training were identified by participants as reduced stress for animals and personnel, better study data, and improved animal welfare. Some of the other common answers were related to animals being easier to handle, personnel being happier, improved job efficiency, and reduced fear and aggression from the animals. When asked what the concerns were with using these techniques, half of the participants stated they had no concerns. However, when concerns were mentioned, the top responses were related to training personnel to use the techniques properly and consistently, the time needed to implement these techniques, and expectations for negative researcher attitudes. Other concerns included the potential cost for purchasing tunnels to use for handling, the applicability of the techniques across research projects, the number of animals that would need to be trained, and animal health or biosecurity issues associated with direct animal handling. An interesting key quote spoke to the need to minimize the use of these techniques to help personnel maintain emotional and physical distance from research animals as a means of avoiding potential compassion stress/fatigue:

“People get very attached to the animals—there is potential of compassion fatigue.”

There were differences in responses between countries and job areas. For benefits related to low stress handling, CA participants were more likely to mention improved animal welfare as the top benefit. DL respondents were more likely to say that the benefits would include reduced stress and improved data, compared to NDL. In terms of concerns for using low stress handling techniques, NDL were more likely to say there were no concerns about using these techniques but for DL the need for training personnel was the top concern. Between countries, US participants were more concerned about the potential for negative researcher attitudes than CA participants.

Most participants were familiar or somewhat familiar with low stress handling techniques with rats and mice, but more CA participants (64.7%) stated “yes” than US participants (56.5%). When asked how they felt about using these techniques or training others to use them, CA participants were more in support of using and training with these techniques, with many saying they already use these techniques or were starting to implement them. US participants were more likely to say they supported using the techniques but indicated concerns such as researcher buy-in, time or that the techniques would not work for all areas of research.

Participants were asked how using these low stress handling techniques in a more widespread fashion would impact their job. The responses were organized by positive and negative impacts. The top positive impact of broadly implementing low stress handling techniques was thought to be improved job satisfaction, with respondents indicating that their job would become easier. Another top benefit was thought to be decreased stress for personnel and animals. For negative impacts, participants believed these methods would take more time and that training personnel would be challenging. There were similar responses across demographic groups. One key quote from this question was:

“I think the biggest concern people have that I work with is it would take more time. But I think in the long run it would decrease stress on everyone.”

Despite many participants seeing time as an issue in using these techniques, 90% of participants agreed it would be worth the time, with the remaining participants saying it would be worth the time but they still expressed concerns about how much time it would take and the likelihood of negative researcher attitudes. NDL were more likely to state concerns than DL. When asked if the researchers would think it was worth the time, most participants said no or that it would depend on the specific researcher.

### Video responses and implementation

Most participants had not seen the nonaversive handling videos of mice or rats prior to the interview. The responses to the videos were overwhelmingly positive, such as:

“I think it's pretty amazing that just with a couple sessions that you can use low stress handling techniques on a rat or mouse.”

“I thought it was very interesting. I saw a lot of novel techniques that I never heard of before, such as clicker training with rodents. I'm curious to see how that would work in my research environment.”

Participants were also largely surprised to see low stress handling techniques specifically for mice:

“I was surprised by the mice, because I have more of the opinion that mice did not want to interact with humans as they are more fearful compared to rats.”

A common response to the videos from DL participants was that they wanted to implement the techniques at their facility and they mentioned action items, such as intending to send emails to people within their facility after the interview.

Following viewing of the videos, participants would still mention positive opinions followed by concerns such as the number of rodents at their facility reflected in these quotes:

“…might be difficult to scale up to the size of a very large university”

“…may not be very practical when you're dealing with much larger quantities of rodents”

Or stating challenges of getting buy-in at their facility:

“…there are PIs I have to convince on things. I'd be doing it yesterday if I had approval.”

US participants were more likely to have their opinions on low stress handling changed as a result of watching the videos while CA participants were more likely to indicate that the videos further supported their knowledge, such as:

“No, I was already on board, we're already doing some of this.”

Despite that US participants indicated that their opinions had changed as a result of viewing the videos, they were also more skeptical or hesitant of the techniques demonstrated, continuing to state challenges and impediments they would face in using these techniques, and this was especially true for US NDL. When asked if the techniques were possible at their facility, 40% indicated “yes”, 35.5% indicated “no”, and the remaining respondents indicated that they were unsure or that it would be possible for some experiments but not all of them. For participantsstating “yes” and “no”, the same concerns were mentioned. One participant stated that “yes, it was possible”, but added: “Yes, but not for a long time.” The primary perceived barriers to implementation are presented in Figure 2. The top barriers indicated were lack of time, money, or resources, and lack of researcher support or negative researcher attitudes. Additional concerns and barriers suggested were that these techniques couldn't be used for some types of research, there were too many animals that needed to be trained, training personnel would be difficult, and that there needed to be more evidence supporting the benefits of nonaversive handling of mice and rats. In general, there were similar concerns across demographics groups, but US participants were more likely to mention negative researcher attitudes or type of facility or research as barriers, and NDL in both the USA and CA were more uncertain if these techniques could be implemented at their facility.

## Discussion

The goal of this study was to explore the understanding, experiences, and attitudes of laboratory animal professionals in the USA and Canada toward low stress handling, habituation, and basic training techniques with rats and mice. While the participants of the study were supportive of using these techniques, there was limited understanding and experience with using the techniques specifically with rats and mice. There were also a number of perceived barriers to implementation, including anticipated negative researcher attitudes, the time or resources required to implement and use these techniques, and the time needed to train personnel.

Participants in this study recognized the benefits of using low stress handling and training techniques, but the techniques were not widely implemented at many of the represented facilities at the time of the interviews. It was common for participants to mention using these techniques with other species but many were surprised to see them being used with rats and mice, particularly mice, suggesting species-specific attitudes impeding refinements. Speciesism, or lower moral, ethical or welfare consideration of some species compared to others (30), is often apparent for research rodents compared to other species such as primates and dogs (31). Reasons cited for this include the degree of phylogenetic or behavioral proximity to peoples, degree of animal self-awareness, perceived bonding or relationship with people, intelligence, perceived sensitivity to pain or discomfort, and cognitive or behavioral complexity of the animal (31). Despite this, it is well known that rats and mice are intelligent and sensitive animals who respond quickly to habituation and training (32–35). Further education and research opportunities should focus on demonstrating how these techniques could be incorporated for rats and mice on a large scale and with different types of research.

In the current study, some participants mentioned an apparent lack of evidence that these techniques are beneficial as a reason for not implementing low stress handling. In a study by Henderson et al. (28), respondents questioned the methodology of past studies in relation to practical applications. Respondents also stated that most research highlights the behavioral outcomes, and there needs to be more evidence on the physiologic outcomes, particularly related to stress physiology, cardiovascular indicators, and health outcomes related to surgery, anesthesia, drug delivery, and oncology (28). In mice, there is over a decade of research demonstrating the behavioral and physiologic benefits of low stress handling. Besides the benefits to animal welfare, low stress handling methods improve operational outcomes such as breeding productivity. Hull et al. (14) demonstrated that tunnel handling resulted in more pups born and weaned per litter, with a 20% reduced risk of litter loss compared to when dams were handled by their tail. Low stress handling also improves research outcomes by reducing variation in the study data (17), particularly for disease models that would be susceptible to stressors (13). Effects of handling have also been reflected in physiologic measures for plasma corticosterone (12, 13) and blood glucose levels (12). Empirical evidence continues to demonstrate the effects of handling procedures on health, welfare, and research outcomes but has focused primarily on mice. Fewer studies have asked similar research questions with rats, leaving a knowledge gap on specific preferred handling methods and implementation.

Time to implement and use low stress handling was mentioned as a significant perceived barrier to implementation in this study and in a previous study (28). Past research has demonstrated that it takes as little as 2s per handling session at routine cage change to see the benefits of low stress handling for mice (19). There is evidence that the process of training personnel to use low stress handling techniques initially may result in increased time to cage change compared to using tail handling with the hand or forceps (36), but this diminishes as caregivers become more skilled with the technique (14). Exclusively using this method during personnel training would also speed proficiency and uptake vs. retraining personnel on a basic skill that they may have become accustomed to using for years to decades. Positive human-animal interactions can act as a reward during operant conditioning for rats (37), further emphasizing possible benefits of this technique. Positive interactions with animals is also beneficial for human mental health and job satisfaction (38–40).

One of the most common barriers mentioned was a perceived lack of researcher support to implement these techniques. Researcher support is vital to implementing changes to research practices (31). Survey results from Henderson et al. (28) indicated that researchers were more likely to use tail handling only and that they hadn't heard about nonaversive (tunnel) handling compared to laboratory animal professionals. In that work, when asked for reasons as to why tunnel handling wasn't used, common answers were that they were using the handling methods that have always been used, they had not heard about tunnel handling or no one had suggested they do it differently (28). Participants in that survey also stated there would need to be a top-down approach to see any change in handling method, as resources need to be provided to obtain tunnels as well as training on how to use them (28). In general, researchers may be lacking in knowledge related to optimal animal welfare practices and the 3Rs. For example, only 20% of FELASA researchers surveyed in 2014 were able to correctly name and define the 3Rs principles (31). In this study and in the results presented by Henderson et al. (28), laboratory animal professionals were receptive when shown these methods. Better outreach on the benefits of low stress handling methods needs to be directed at all levels within research community to build awareness and consensus. Providing training on topics related to the 3Rs and animal welfare can help improve knowledge of 3Rs principles and encourage researchers to implement 3Rs practices in their laboratories (31). Training on low stress handling, habituation, and training practices for rats and mice geared toward all levels, but particularly researchers, could encourage more facilities in the US and Canada to adopt these techniques.

An important concern, particularly from nondoctoral level personnel in this study, was a perceived inability to influence or drive change in practices at their institutions. A key component of a Culture of Care is being able to have a positive attitude toward animal welfare initiatives as well as the ability to be open to change. This includes providing support to listen to, consider, and pilot ideas from all employees, and then to act on and implement validated changes when viable (41). From the responses provided in this study, technical personnel commonly predicted negative responses to new proposed animal welfare initiatives, and they did not feel empowered to drive changes at their respective institution. Ensuring that employees at all levels feel valued and that they can bring new ideas and concerns forward are important elements of an institution's Culture of Care as well as keeping up-to-date with the 3Rs in research settings, a societal imperative (41).

There were limitations of the study particularly related to response bias. Participation was voluntary based on response to email advertisements. This resulted in gender and country imbalance with most respondents being female from the USA. There is evidence of gender differences in attitudes toward animals, with females generally having more positive attitudes toward animals and indicating a greater concern for animal welfare than males (42). Male handlers may also induce a stress response in animals, such as rodents, which may impact observed animal behavior and welfare (43). This suggests that there may be less overall awareness about the benefits of low stress handling techniques as well as training for mice and rats amongst the wider population of laboratory animal professionals. The nature of an interview study may also introduce bias regarding participants based on individual time constraints, personality traits (introverts vs. extraverts), concerns about anonymity, access to a computer to participate in the interview, and other factors that can result in participants having similar backgrounds and views. Participants who volunteered may already be concerned about handling methods in rodents and may have an interest in low stress handling. As a result, the views presented in this paper likely are not fully representative of attitudes across laboratory animal professionals but can hopefully provide insight into how to move forward with promoting low stress handling.

In conclusion, laboratory animal professionals interviewed from the USA and Canada understand the benefits of low stress handling techniques, but despite this, have not moved toward widespread adoption for research mice and rats. There was also limited knowledge and experience with low stress handling, habituation, and training techniques, and a lack of evidence was cited as a concern for implementing these techniques, despite numerous publications from the past 10 years demonstrating the benefits, particularly with mice. This suggests a gap in knowledge and experience that should be addressed for personnel working with research rodents at all levels. Negative researcher attitudes were anticipated to be the primary barrier preventing implementation of nonaversive handling and training techniques for mice and rats, suggesting that resources and training need to be developed and promoted for this group to encourage implementation of the 3Rs.

## Data availability statement

The raw data supporting the conclusions of this article will be made available by the authors, without undue reservation.

## Ethics statement

The studies involving human participants were reviewed and approved by University of Guelph Research Ethics Board (REB#: 20-06-022). Written informed consent for participation was not required for this study in accordance with the national legislation and the institutional requirements.

## Author contributions

CM and PT: conceptualization and design. CO'M and RH: data collection and analyses. CO'M and PT: writing and editing manuscript. All authors contributed to the article and approved the submitted version.

## References

[1] Mellor DJ Beausoleil NJ Littlewood KE McLean AN McGreevy PD Jones B. The 2020 five domains model: including human-animal interactions in assessments of animal welfare. Animals. (2020) 10:1870. 10.3390/ani10101870 33066335 PMC7602120

[2] LaFollette M. Human-Animal Interactions. In Sørensen DB Cloutier S Gaskill BN (eds). Animal-Centric Care and Management: Enhancing Refinement in Biomedical Research. Boca Raton, FL: CRC Press (2021). p. 1–13.

[3] Gouveia K Hurst JL. Improving the practicality of using non-aversive handling methods to reduce background stress and anxiety in laboratory mice. Sci Rep. (2019) 9:20305. 10.1038/s41598-019-56860-7 31889107 PMC6937263

[4] Rault J-L Wainlinger S Boivin X Hemsworth P. The power of a positive human-animal relationship for animal welfare. Front Vet Sci. (2020) 7:590867. 10.3389/fvets.2020.590867 33240961 PMC7680732

[5] Rankin CH Abrams T Barry RJ Bhatnagar S Clayton DF Colombo J. Habituation revisited: an updated and revised description of the behavioral characteristics of habituation. Neurobiol Learn Mem. (2009) 92:135–8. 10.1016/j.nlm.2008.09.012 18854219 PMC2754195

[6] Sørensen DB Pedersen A Forkman B. “Animal Learning: The Science behind Animal Training”. In Sørensen DB Cloutier S Gaskill BN (eds). Animal-Centric Care and Management: Enhancing Refinement in Biomedical Research. Boca Raton, FL: CRC Press (2021). p. 59–71.

[7] Hickman DL Johnson J Vemulapalli TH Crisler JR Shepherd R. “Commonly Used Animal Models.” In Suckow M, Stewart K (eds). Principles of Animal Research for Graduate and Undergraduate Students. Cambridge, MA: Academic Press (2016). p. 117–75. 10.1016/B978-0-12-802151-4.00007-4

[8] Adams KM Navarro AM Hutchinson EK Weed JL A. Canine socialization and training program at the national institutes of health. Lab Anim. (2004) 33:32–6. 10.1038/laban0104-32 14752529

[9] Meunier LD. Selection, acclimation, training, and preparation of dogs for the research setting. ILAR J. (2006) 47:326–47. 10.1093/ilar.47.4.326 16963813

[10] Westlund K. Training laboratory primates—benefits and techniques. Primate Biol. (2015) 2:119–32. 10.5194/pb-2-119-2015

[11] Hurst JL West RS. Taming anxiety in laboratory mice. Nat Methods. (2010) 7:825–6. 10.1038/nmeth.1500 20835246

[12] Ghosal S Nunley A Mahbod P Lewis AG Smith EP Tong J. Mouse handling limits the impact of stress on metabolic endpoints. Physiol Behav. (2015) 150:31–7. 10.1016/j.physbeh.2015.06.021 26079207 PMC4546855

[13] Ono M Sasaki H Nagasaki K Torgoe D Ichii O Saski N. Does the routine handling affect the phenotype of disease model mice? Jpn J Vet Res. (2016) 64:265–71. 10.14943/jjvr.64.4.265 29786176

[14] Hull MA Reynolds PS Nunamaker EA. Effects of non-aversive versus tail-lift handling on breeding productivity in a C57BL6J mouse colony. PLoS ONE. (2022) 17:e0263192. 10.1371/journal.pone.0263192 35089969 PMC8797240

[15] Gouveia K Hurst JL. Reducing mouse anxiety during handling: effect of experience with handling tunnels. PLoS ONE (2013) 8:6. 10.1371/journal.pone.0066401 23840458 PMC3688777

[16] Clarkson JM Leach MC Flecknell PA Rowe C. Negative mood affects the expression of negative but not positive emotions in mice. Proc R Soc B. (2020) 287:20201636. 10.1098/rspb.2020.1636 32842924 PMC7482280

[17] Nakamura Y Suzuki K. Tunnel use facilitates handling of ICR mice and decreased experimental variation. J Vet Med Sci. (2018) 80:886–92. 10.1292/jvms.18-0044 29657231 PMC6021882

[18] Clarkson JM Dwyer DM Flecknell PA Leach MC Rowe C. Handling method alters the hedonic value of reward in laboratory mice. Sci Rep. (2018) 8:2448. 10.1038/s41598-018-20716-3 29402923 PMC5799408

[19] Gouveia K Hurst JL. Optimising reliability of mouse performance in behavioural testing: the major role of non-aversive handling. Sci Rep. (2017) 7:44999. 10.1038/srep44999 28322308 PMC5359560

[20] Henderson LJ Dani B Serrano EMN Smulders TV Roughan JV. Benefits of tunnel handling persist after repeated restraint, injection, and anaesthesia. Sci Rep. (2020) 2:11. 10.1038/s41598-020-71476-y 32884048 PMC7471957

[21] Lorenzini CA Bucherelli C Giachetti A Tassoni G. Inhibition of exploratory behavior in the rat by handling. Anim Learn Behav. (1990) 18:191–8. 10.3758/BF032052587960495

[22] Costa R Tamascia ML Nogueira MD Casarini DE Marcondes FK. Handling of adolescent rats improves learning and memory and decreases anxiety. JAALAS. (2012) 51:548–53. 23312082 PMC3447442

[23] Manseur A Bairi A Bakeche A Djouini A Tahraoui A. Effect of handling by human being neonatal period on anxiety and depression-like behavior of adult rats. Adv Anim Vet Sci. (2019) 7:1113–9. 10.17582/journal.aavs/2019/7.12.1113.11192019

[24] Hirsjärvi PA Junnila MA Väliaho TU. Gentled and non-handled rats in a stressful open-field situation; differences in performance. Scand J Psychol. (1990) 31:259–65. 10.1111/j.1467-9450.1990.tb00838.x 2274759

[25] Maurer BM Döring D Scheipl F Küchenhoff H Erhard MH. Effects of a gentling programme on the behaviour of laboratory rats towards humans. Appl Anim Behav Sci. (2008) 114:554–71. 10.1016/j.applanim.2008.04.013

[26] Stuart SA Robinson ESJ. Reducing the stress of drug administration: implications for the 3Rs. Sci Rep. (2015) 5:14288. 10.1038/srep14288 26395864 PMC4585806

[27] Russel WMS Burch RL. The Principles of Humane Experimental Technique. London; UFAW: Potters Bar; Herts: Methuen. (1959). 239 p.

[28] Henderson LJ Smulders TV Roughan JV. Identifying obstacles preventing the uptake of tunnel handling methods for laboratory mice: an international thematic survey. PLoS ONE. (2020) 15:e0231454. 10.1371/journal.pone.0231454 32287297 PMC7156035

[29] Kvale S Brinkmann S. Interviews: Learning the Craft of Qualitative Research Interviewing. Thousand Oaks, CA: SAGE Publications, Inc. (2009).

[30] Horta O Albersmeier F. Defining speciesism. Philos Compass. (2020) 15:e12708. 10.1111/phc3.12708

[31] Franco NH Olsson IAS. Scientists and the 3Rs: attitudes to animal use in biomedical research and the effect of mandatory training in laboratory animal science. Lab Anim. (2014) 48:50–60. 10.1177/0023677213498717 23940123

[32] Poling A Weetjens B Cox X Beyene NW Bach H Sully A. Using trained pouched rats to detect land mines: another victory for operant conditioning. JABA. (2013) 44:351–5. 10.1901/jaba.2011.44-351 21709791 PMC3120071

[33] Leidinger C Herrmann F Thone-Reineke C Baumgart N Baumgart J. Introducing clicker training as a cognitive enrichment for laboratory mice. J Vis Exp. (2017) 3:e55415. 10.3791/55415 28287586 PMC5408971

[34] Leidinger CS Kaiser N Baumgart N Baumgart J. Using clicker training and social observation to teach rats to voluntarily change cages. J Vis Exp. (2018) 140:e58511. 10.3791/58511 30417890 PMC6235608

[35] Crawford LE Knouse LE Kent M Vavra D Harding O LeServe D. Enriched environment exposure accelerates rodent driving skills. Behav Brain Res. (2020) 378:112309. 10.1016/j.bbr.2019.112309 31629004

[36] Doerning CM Thurston SE Villano JS Kaska CL Vozheiko TD Soleimanpour SA. Assessment of mouse handling techniques during cage changing. JAALAS. (2019) 58:767–73. 10.30802/AALAS-JAALAS-19-000015 31645236 PMC6926398

[37] Davis H Pérusse R. Human-based social interaction can reward a rat's behavior. Anim Learn Behav. (1988) 16:89–92. 10.3758/BF03209048

[38] Beetz A Uvnäs-Moberg K Julius H Kotrschal K. Psychosocial and psychophysiological effects of human-animal interactions: the possible role of oxytocin. Front Psychol. (2012) 3:1–15. 10.3389/fpsyg.2012.00234 22866043 PMC3408111

[39] Davis ME. Effect of Human-Animal Interactions on Retail Employees' Job Satisfaction and Job Performance [dissertation]. Minneapolis, MN: Walden University.

[40] Randall MS Moody CM Turner PV. Mental wellbeing in laboratory animal professionals: a cross-sectional study of compassion fatigue, contributing factors, and coping mechanisms. J Am Assoc Lab Anim Sci. (2021) 60:54–63. 10.30802/AALAS-JAALAS-20-000039 33028460 PMC7831355

[41] Bertelsen T Øvlisen K. Assessment of the Culture of Care working with laboratory animals by using a comprehensive survey tool. Lab Anim. (2021) 0:1–9. 10.1177/00236772211014433 34039088

[42] Herzog HA. Gender differences in human-animal interactions: a review. Anthrozoös. (2007) 20:7–21. 10.2752/089279307780216687

[43] Sorge RE Martin LJ Isbester KA Sotocinal SG Rosen S Tuttle AH. Olfactory exposure to males, including men, causes stress and related analgesia in rodents. Nat Meth. (2014) 11:629–32. 10.1038/nmeth.2935 24776635

