# Supplementary material for: Use of nonaversive handling and training procedures for laboratory mice and rats: Attitudes of American and Canadian laboratory animal professionals
Source: Front Vet Sci. 2022 Dec 9;9:1040572. doi: 10.3389/fvets.2022.1040572 (PMC9780379; doi:10.3389/fvets.2022.1040572)
Supplement: Supplementary file 1 [file Data_Sheet_1.docx]

**Interview Questions:**

1. As a laboratory animal science professional, what is your single biggest day to day concern while interacting with research mice and/or rats?
2. What is your understanding of the terms: habituation and positive reinforcement training?
3. What is your experience in using habituation and positive reinforcement training with laboratory mice and rats?
   1. What are the potential benefits of using habituation and positive reinforcement training with laboratory mice and rats?
   2. Do you have concerns about using habituation and positive reinforcement training with laboratory mice and rats?

The next few questions are related to handling mice and rats.

1. Are you familiar with low stress handling techniques that can be used for mice and rats?
2. How do you feel about using these/training others to use these types of handling techniques with laboratory mice and/or rats?
3. Do you see any connection between the use of low stress handling techniques and use of habituation and positive reinforcement training with mice and rats?
4. How would using low stress handling as well as habituation and positive reinforcement training techniques impact your job?
   1. Do you think it’s worth it to add time to incorporating these methods into your daily routine?
   2. Do you think that researchers at your institution find it worth it to include these techniques?

*Participant watches RiSe Mouse and rat handling/training videos:*

*Mouse:* <https://www.youtube.com/watch?v=bdtVZtrr69c&feature=emb_logo>

*Rat:* <https://www.youtube.com/watch?v=gbsz_LZwuCM&feature=emb_logo>

1. Are you familiar with these videos?
2. What do you think about the handling and training techniques used in the 2 videos?
3. After watching these videos, has your perspective changed about adding time to incorporate low stress handling as well as habituation and positive reinforcement training procedures into your daily routine or into your institutional training procedures?
   1. Do you think this would be possible at your facility? Why or why not?
4. Is there anything else related to rodent handling or rodent training that you’d like to mention or discuss?
